# Application and Advances in Radiographic and Novel Technologies Used for Non-Intrusive Object Inspection

**DOI:** 10.3390/s22062121

**Published:** 2022-03-09

**Authors:** Dmytro Mamchur, Janis Peksa, Soledad Le Clainche, Ricardo Vinuesa

**Affiliations:** 1 Information Technologies Department, Turiba University, Graudu Street 68, LV-1058 Riga, Latvia; 2 Computer Engineering and Electronics Department, Kremenchuk Mykhailo Ostrohradskyi National University, Pershotravneva 20, 39600 Kremenchuk, Ukraine; 3 Institute of Information Technology, Riga Technical University, Kalku Street 1, LV-1658 Riga, Latvia; janis.peksa@rtu.lv; 4 School of Aerospace Engineering, Universidad Politécnica de Madrid, 28040 Madrid, Spain; soledad.leclainche@upm.es; 5 FLOW, Engineering Mechanics, KTH Royal Institute of Technology, SE-100 44 Stockholm, Sweden

**Keywords:** X-ray, biosensor, artificial intelligence, classification, non-intrusive inspection

## Abstract

Increase in trading and travelling flows has resulted in the need for non-intrusive object inspection and identification methods. Traditional techniques proved to be effective for decades; however, with the latest advances in technology, the intruder can implement more sophisticated methods to bypass inspection points control techniques. The present study provides an overview of the existing and developing techniques for non-intrusive inspection control, current research trends, and future challenges in the field. Both traditional and developing methods, techniques, and technologies were analyzed with the use of traditional and novel sensor types. Finally, it was concluded that the improvement of non-intrusive inspection experience could be gained with the additional use of novel types of sensors (such as biosensors) combined with traditional techniques (X-ray inspection).

## 1. Introduction

Worldwide globalization processes aim to make the world more open offering the possibility of easy access to different places around the world regardless of their citizenship or nationality. It also helps make it possible to choose places to visit, to live, and to work based on one’s own preferences without any obstacles during travelling [1,2,3,4,5,6,7,8,9]. Additionally, the modern world with its global integration and interconnection of all processes extremely depends on trading. Additionally, to make trading more effective, it is desirable to reduce trading barriers, including time-consuming bureaucratic and legal procedures at a customs control point [1].

However, this openness is often exploited by different categories of malefactors intent upon spreading illicit or dangerous goods and materials, including smuggling, weapons, narcotics, etc., around the globe. Moreover, with advances in IT, electronics, and other modern technologies, the ways and media employed by criminals to bypass legal control procedures are becoming more and more sophisticated.

After the EU migration crisis in 2015, several states from the Schengen group temporarily restored control at their national borders [3,4,5,6,7,8]. However, this created additional obstacles for travelers and trading companies, and could not be considered as an effective countermeasure for economic reasons [3,4,5,6,7,8]. Thus, different approaches to increasing national security, along with improvement of border control experience both for travelers and trading companies, providing comprehensive but expeditious non-intrusive inspection, should be applied.

In order to counter these constantly growing threats, governmental and intergovernmental organizations establish specialized agencies, implement novel automated inspection technologies at customs and other control points, and issue improved instructions for non-intrusive inspection mechanisms. However, big international criminal organizations still remain ahead of security control establishments with the use of both legitimate and illegitimate supply chains exploiting more advanced technology and instruments in bypassing the inspection process. So, the question remains, whether the currently existent inspection mechanism provides an adequate level of security and protection for the society and effectively helps to reduce the illegal trade level. Thus, there is still a need for cooperation and information exchange between different branches of the security control process, as well as a strong demand for the development of novel techniques and technologies for check point inspection control.

Over the last few decades, governments in different parts of the world have been introducing novel instruments in inspection, particularly, in border control management. For example, a European Border and Coast Control Agency, known as Frontex was established within the European Union’s Schengen Area with the aim of coordinating border control efforts at the external borders of the European Union [10]. The USA constantly improves protection on the border control points with Mexico, where they have a constant issue with illegal migration and smuggling [11]. Similar challenges are being faced in other parts of the world, such as Oceania, Asia, Africa, and South America [11]. In each area, governments are taking measures, which could be different in nature and dependent on typical regional peculiarities, such as continuous integration of EU member countries in Europe, geographical challenges in Oceania and North America, and historical challenges in Africa and South America [11].

These novel techniques for improvement of border control experience for legitimate travelers typically relate to person identification technologies based on behavioral or physical characteristics and include automated methods to recognize fingerprints, hand and finger geometry, retinal and iris scanning, voice and facial recognition [12,13]. As an example, over the last 15 years SmartGate, ePassport, and eVisitor technologies are being successfully implemented and exploited in Australia [14]. Working together, these technologies using information from the traveler’s biometric passport (such as name, date of birth, and photograph), using facial recognition technologies and information from the national database (such as banned travelers database) to automatically verify a person’s identity and make a decision on whether to grant entry to the country, or allow the person to depart, or send the person to a customs official for additional manual inspection. Similarly, the USA established US-VISIT Program (US Visitor and Immigrant Status Indicator Technology) [15], which provides border control points with biometric technology helping to verify the visiting person’s identity based on facial and fingerprint recognition techniques as well as using verification through available databases.

Bad experiences with the implementation of such technologies should also be mentioned. Thus, in 2007, the UK brought in an e-Border project, aimed to collect and store information on passengers and crew entering and leaving the country [16]. However, in 2014 the project was terminated due to constant delays in its implementation [16]. This example allows us to testify that the problem of border control automation and improvement security throughout the control points remains complex and requires improvement in the approaches considered to its solution, involving close cooperation of different types of organizations and institutions, including legislative, governmental, intergovernmental, and technical ones as well.

Thus, the evolution of security control at national borders has resulted in an increasing number of border control types, the amount of data to be controlled, and the number of technologies used to provide all these types of control [11]. Multiplication in technologies used for border control results in the implementation of novel sensors, hardware, and software, as well as data analysis systems for surveillance, identification, data storage, and analysis.

Surveillance technologies have constantly evolved during the past 20–30 years. They include X-ray screening techniques for non-intrusive inspection of baggage, personal belongings, cargo, etc., non-intrusive body inspection techniques, air analysis techniques to detect explosives, narcotics, people that might be hidden in cargo, and other illicit goods [12,13].

One of the most important challenges in non-intrusive inspection is the dataflow between different systems and different governments. Over the past 20 years, several integrated data systems which are still regularly enhanced, have been introduced in Europe.

The most widely known in Europe are the following:

- The Schengen Information System (SIS). This system takes its roots from 2001, and currently coordinates the databases of 26 countries. Technically, SIS is based on classic star data processing architecture, with the reference database located in the French Republic, while all other states have a copy of this database. SIS stores data on persons that are refused entry; suspected of a crime; data referred to lost or stolen weapons, motor vehicles, identity documents, etc. France manages the SIS reference database and is responsible for its constant update every five minutes [17].
- Eurodac. European Asylum Dactyloscopy Database (Eurodac) is a database that includes fingerprints of asylum seekers and irregular border crossers [18]. This database is used by 27 EU Member States and Associated Countries and is aimed at controlling both the legal and illegal movement of asylum seekers within Eurodac countries.
- The Visa Information System (VIS). This database contains information required from visa applicants wishing to enter Schengen Area countries, including biometric data, such as fingerprint and facial images. Similar systems are implemented in other parts of the globe, namely, in the USA it is known as the Arrival and Departure Information System (ADIS) [19], SmartGate in Australia, and others [20].

These examples demonstrate the possibility of deep integration of the information systems across different states and combining them into a united database available to each participating state or organization. All these examples mostly relate to person identification issues; however, the question of items identification, development of similar databases containing data on legal and illegal items, remains open. Additionally, the problem of a comprehensive non-intrusive inspection at control checkpoints which provides fast and highly reliable results with detection of the most typical threat types, and does not significantly delay people and cargo flows, still needs deeper improvement. The following chapters reveal traditional and developing techniques, which could be enhanced for the aims of non-intrusive object inspection.

## 2. Technology Used for X-ray Screening

The most popular technique, currently used at almost every airport and several control points for quick non-intrusive inspection, is X-ray screening. There are several different X-ray scanning techniques, which differ by their complexity, cost, and results provided. Currently, two main groups of X-ray machines based on either single- or dual-energy analysis, are most widely used [21,22,23]. Simple single-energy machines provide black-and-white 2D images, representing the “shadow” of an inspected object generated by direct X-rays penetration through it. These images could provide general information about the object’s shape and density, but information about the object’s depth and structure remain unknown; thus, additional image processing techniques need to be applied for the subsequent processing of the retrieved raw information.

Dual-energy machines generate high- and low-energy X-ray beams as a result of a Compton effect and use the scattered radiation analysis to determine atomic numbers of the inspected object. These atomic numbers are subsequently used for object material determination, helping to recognize and classify objects both by their shape and composition.

Recently, a number of more advanced techniques have appeared, but all of them use the basic principles of X-ray inspection and are described below.

### 2.1. Planar Radiography

One of the most popular and widely used solutions for X-ray inspection is based on the planar radiography principle. Within this technology, single-energy X-ray machines are used to generate X-radiation beams that penetrate inspected objects. Different types of materials, depending on their density, absorb different percentages of X-ray radiation, which results in a different shade of gray on the resulting image: material with a high-density structure, such as bones and metals, absorb a higher percentage of radiation and appear lighter in the resulting image, while low-density materials could absorb nothing, and appear black in the resulting picture (e.g., air). To record an image, the receptor is placed opposite to the X-ray source, and the inspected object appears between the source and detector.

**Figure 1:**
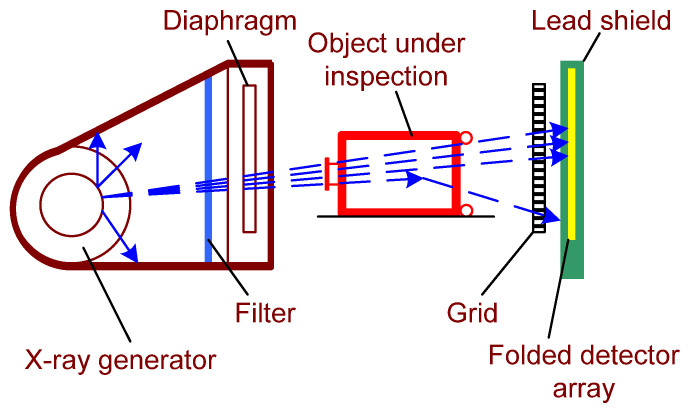
Schematic of a standard projection radiography setup used for non-intrusive inspection of a large-sized item.

The typical schematic of a standard planar radiography setup is presented in Figure 1.

A typical challenge in planar radiography is the superposition of compositional variations within the object under inspection. To solve this issue, several methods for determination of the object’s depth were developed. One such method proposes to use the rotation of the coordinates to obtain additional features which could be used to determine the depth of the object. This approach assumes the creation of two orthogonal images from frontal and lateral views.

**Figure 2:**
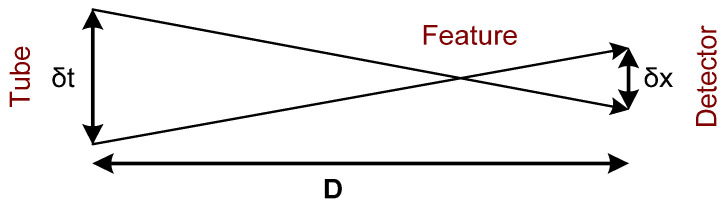
The idea of a rectilinear tomographic scanning system aiming to ensure the blur out of projection details.

Another approach proposes the creation of two images displaced by a few degrees, which could be then viewed in a stereoscope to get a full picture of an object under inspection (Figure 2). With this technique, it should be considered that while focus and detector move, the images of all points in a plane under consideration should be in the same set of points within the detector plane.

Planar radiography systems could mainly provide information about the object’s shape. However, it is not easy to obtain quantitative information about the object’s density due to the presence of interfering materials within the object under inspection (e.g., bag or package).

### 2.2. X-ray Computed Tomography (CT)

Another special radiographic technique is tomography. This technique was introduced with the aim of overcoming the overlapping issue by rotating coordinates, where the image detector and the X-ray tube continuously move in opposite directions over a limited range [24]. Only the elements in the plane containing the fulcrum remain stationary in this technique, while the smoothness of movement blurs out the image of other object components outside the fixed layer. The movement angle and required resolution form the thickness of the section in focus within this method.

With advances in digital computer performance, it became possible to employ them, in combination with counters based on ionization or scintillation principles, to X-ray radiography tasks. This allowed imaging environments with a low atomic number, such as soft tissues, which ignited a novel era in clinical diagnostics, and later in material control and detection techniques.

**Figure 3:**
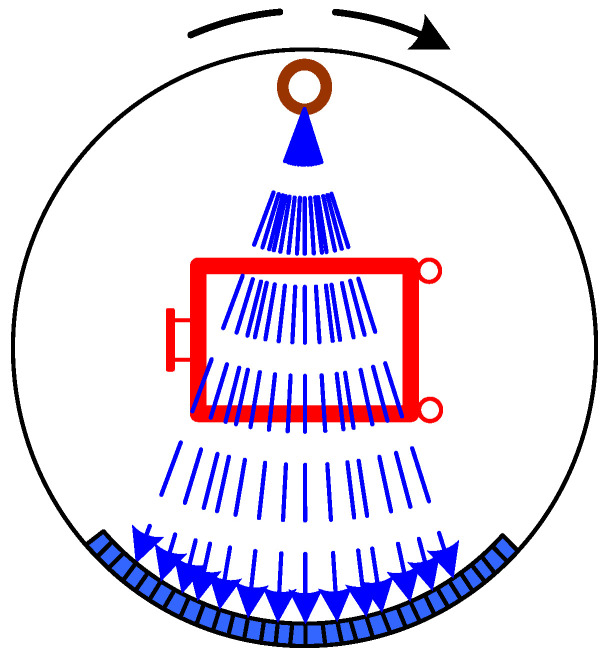
Schematic representation of a Real-Time Computed Tomography device.

Within this technique, a narrow X-radiation beam from a rotating pencil passes through the object under inspection, and then through a carefully aligned collimator to be received by a detector array (Figure 3). Such a technique allows one to overcome the problem of overlapping and provides the possibility of detecting variations in the linear attenuation coefficients for low-nuclear media as low as 0.05% [24]. This capability is due to the possibility of electronically spreading the range of numbers corresponding to the typical attenuations for different media along a beam direction (also called windowing). The detector’s output then connects to a fast computer which provides the final image representation. Such an approach allows one to obtain a comprehensively inspected object representation, including its shape and depth, solving superposition issues. Such technology, for example, proves to be effectively used for the detection of some types of explosives during border control inspection [25].

### 2.3. Dual- and Multi-Energy Imaging

As mentioned in [25], CT scanners used for explosives detection at the border control points are closely similar to medical CT scanners based on single- and dual-energy multi-slicing. Such an approach allows one to eliminate mechanical rotation of a gantry and simplify the construction of a CT machine. Additionally, this research suggested reducing the number of views needed for successful material and shape recognition, which reduces device cost while retaining the possibility of providing reliable detection of both shape and density of an object, which, in its turn, are important features for the detection of harmless and threatening material.

**Figure 4:**
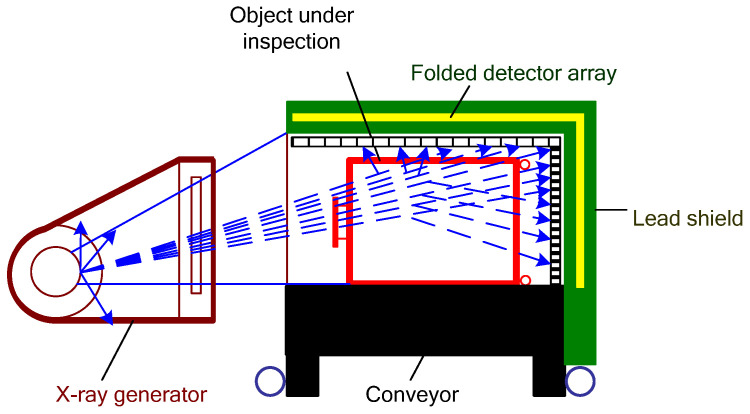
Schematic representation of a combined computed tomography system with a multiple energy X-ray source.

Dual-energy X-ray machines use twice the amount of detecting modules compared to single-energy ones, as they need to sense both high- and low-energy X-rays [26]. Based on signals from both detector types, the system could compute atomic numbers of the inspected materials for the following determination (mostly, materials could be classified as organic, non-organic, and mixtures). In modern systems, detector arrays are installed inside protected L-shaped panels placed diagonally from the X-ray beam generator. Such construction allows one to reduce physical dimensions of the systems along with the exclusion of “blind” zones, and provide the possibility of scanning an entire section of the machine tunnel screening every single part of the inspected object (Figure 4). X-ray sensors provide weak current signals, which, with the use of Analogue-Digital Converters (ADC) are converted into digital signals and are sent to the computing device. Computing software then processes the information from sensors, creating and analyzing an image with subsequent material classification based on corrected low- and high-energy signals. Additionally, the software may colorize an image with dedicated colors for each detected material type.

**Figure 5:**
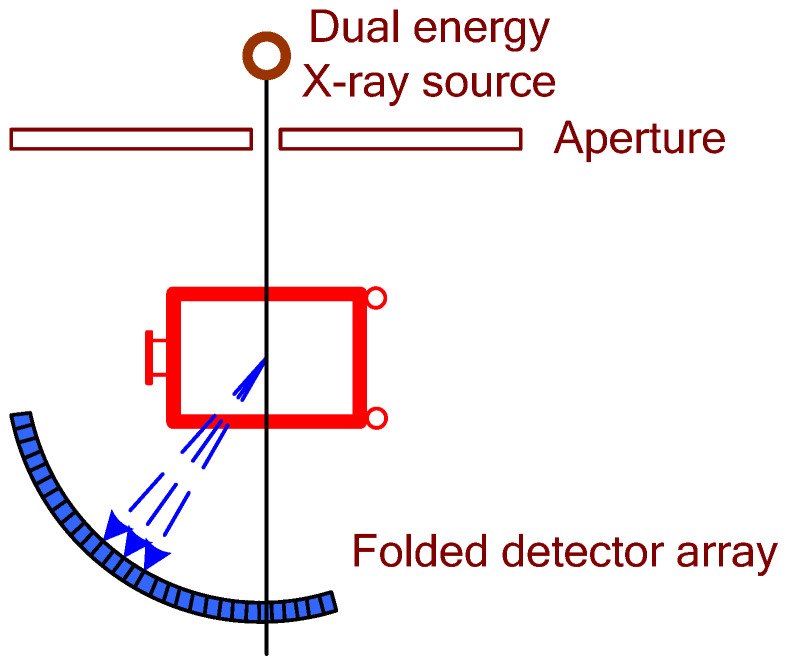
Schematic representation of a multiple energy X-ray computed tomography system.

Multi-energy CT (Figure 5) uses attenuation features of more than two energies within narrow energy ranges; thus, it could provide more physical features of the inspected object and make the inspection more precise and comprehensive [27,28,29]. However, this technique is more expensive as it requires more complex hardware and software for its implementation.

### 2.4. Backscatter Techniques

**Figure 6:**
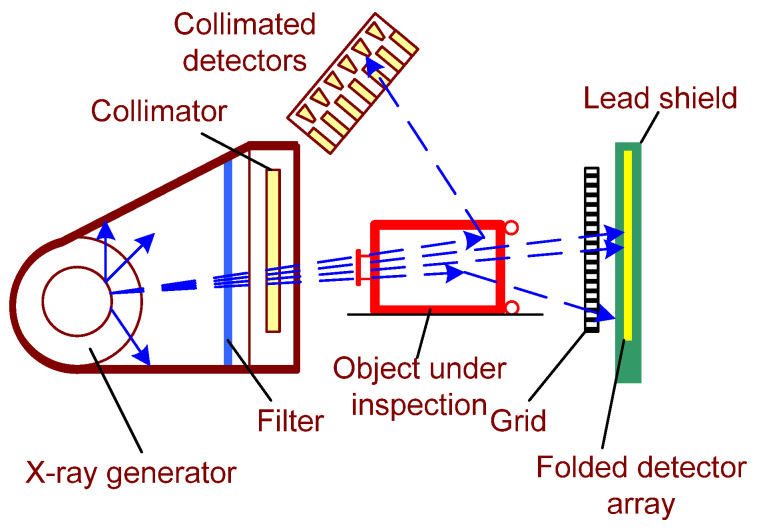
Schematic of a backscattering geometry situation.

In backscatter technology, Compton backscattering effect features are used to build up an inspected object image. In contrast to traditional X-ray techniques which detect the radiation of X-ray passed through the object under study, this technique relies on the detection of the radiation reflected from the object to form an image (Figure 6) [30,31]. The received image shows materials with lower density in a light grey or white color (human skin), while showing higher density materials in a dark grey or black color (firearms). Backscatter X-ray scanners create a 2D image of an object. This technique was successfully employed to search organic materials, such as drugs, explosives, and ceramic weapons.

### 2.5. X-ray Diffraction Imaging

X-ray diffraction (XRD) analysis is a technique used for determining the atomic and molecular structure of a material [32]. This idea implies that the object under study is irradiated with incident X-rays followed by the measurement of scattering angles and the intensity of the X-rays scattered by the material. The molecular structure of the material could be determined by the analysis of such scattered X-ray parameters as their angles, peak positions, and intensity. This technique initially has proved to be effective in liquid medium detection and was later employed for the identification of solid-state explosive materials and drugs in non-intrusive screening machines. However, this approach is currently limited to the identification of organic explosives, and issues concerning the detection of liquid explosives remain open [32].

The analysis of results of current X-ray screening methods and techniques are summarized in Table 1.

### 2.6. X-ray Technology Implementation Use-Cases

All the presented X-ray machine types and principles of X-ray image processing are currently used at different types of control points, starting from airport hand luggage and cargo scanners, ending with postal screening and personal human inspection during public events. Additionally, there are several types of research related to the improvement of the result received with X-ray scanning via modifications in sensor or systems hardware, or raw data processing algorithms. Typical use cases are listed below.

As proposed in [33], a simulation model was constructed using the Monte-Carlo principle for cargo inspection. This model includes a dual-energy source with energy discriminating detectors, which electronically splits pulse flow from detector outputs. Such an approach is a possible alternative to the employment of physical filters (such as lead or copper) for splitting pulse flow. This method allows one to identify such media as tissue-equivalent plastic, silver, aluminum, and iron.

Rapiscan Systems created a screening setup to detect nuclear material in cargo containers [34]. This setup provides two-level screening. The first level, primary screening, uses fast screening based on images received from two independent detector arrays: traditional, primary, detector array, with high spatial resolution, and “rough” energy resolution array. These two detector arrays are used to identify objects with high atomic numbers, such as lead, tungsten, and uranium, which could be used as potential shielding or specialized nuclear materials. The second level is used to recheck container areas with a high atomic number, labeled as suspicious during the first level screening. Secondary screening employs X-ray beams to detect neutron or gamma radiation that could be emitted by the material in the suspicious high atomic number range.

The CT system described in [35] is used for the detection of smuggling in aviation cargo. This system uses multi-axis X-ray screening for fast detection of suspicious cargo. The screening speed is 0.2 m per second, and the time to build a 2-axis image of the full container is up to 40 s.

Ref. [36] proposes the use of the CT system for automatic inspection of the internal structure of complex objects using multiple radioscopies of inspected objects along with various directions and the use of neural network algorithms for data processing. Experimental results for 1000 objects showed less than 4.5% of faulty recognized items.

Authors in [37] have developed a linear electron accelerator to be used as an ionizing radiation source for the customs screening system used for bulky cargo inspection. This device uses a set of collimators at the output of the accelerating device which produces a harp beam of X-rays in a vertical plane. This device allows one to discriminate objects based on organic/non-organic criteria using the dual-energy method.

The experimental results of the practical implementation of a screening CT system for cargo container screening with the aim of detecting special nuclear materials is presented in [38]. The system contains two sets of detector arrays containing 128 detecting elements each, located in parallel and close to one another. The screening speed is 45 cm per second. This CT system has a complex structure which is described in detail in [34]. The experiments showed that the radiation image unevenness of the inspected object significantly influences the different thread detection, which could be explained by the heterogeneity of the object structure.

A novel algorithm for material recognition to be used in baggage control points at the airports and other checkpoints was developed in [39]. This algorithm is based on dual-energy analysis combined with spectral analysis of digital radiation images of the objects under inspection. Experimental results showed the effectiveness of the proposed algorithm to detect metallic, organic, and mixed composition materials with acceptable accuracy and could be used as an operating method.

To assist human operators in the detection of dangerous items hidden in passenger luggage, ref. [40] proposes the automatic items detection and classification based on a combination of multi-view schematic (to obtain X-ray image projections from different angles) and the dual-energy method. This approach was successfully verified experimentally to detect fire guns in hand luggage.

To increase accuracy in the automatic detection of nuclear and explosive materials (e.g., in a seaport container) it was proposed to use three harp-shaped dual-energy X-ray beams [41]. A separate radiation source and set of detector arrays are used to form and detect radiation from every single beam, while central rays from two out of three beams are mutually orthogonal. Athos claims the improvement in operation and reliability of a screening system using such approach.

Different types of CT system construction were proposed in [42], which could be applied for screening cargo containers in variable range sizes, ensuring high inspection efficiency along with acceptable screening speed. In particular, one of the solutions uses multiple screening of the object from different angles.

A general conception of CT system operation for hidden threat detection in aircraft screening was proposed in [43]. These systems typically use backscattering techniques along with the use of gamma rays and neutrons to detect nuclear or radioactive materials.

In [44], the screening CT designed to identify material composition based on its atomic number was described. The system includes at least one X-ray source and detector array operating in spectrometric mode to record the amount of radiation passed through the object under inspection. Along with this equipment, the system could include numerous collimated detectors, which could also operate in spectrometric mode, used to detect backscattering radiation.

The dual-energy system, described in [45] and used in the CT system for personal inspection, uses combined detector arrays, where each detector contains both low- and high-energy detecting elements. As a result of computer processing, the atomic numbers, corresponding to different elements of the object under inspection, are computed. This allows one to discriminate organics, non-organics, and compounds, such as drugs, explosives, and weapons. Additionally, it proposed a novel approach to image processing, which allows one to analyze and collect only body part images with significant differences between detected and normal atomic number values typical for human tissue. This makes the image more convenient for visual inspection, and the screening process is faster and more efficient. Authors claim that such innovation could be widely implemented at airports, custom control points, international bus stations, state administrative buildings, and all the places where non-intrusive and fast screening is needed.

The CT-system for personal inspection in [46] is a dual-energy system with two parallel adjacent detector arrays used for X-ray detection in low- and high-energy zones, respectively.

In [47], a CT system with the use of a high-energy X-ray source used to detect fissile material was described.

Another screening CT based on a multi-view scheme with the use of several X-ray sources for simultaneous screening with several X-ray beams is described in [48].

One of the approaches to enhance the quality of material determination with the use of CT is based on the combination of the dual-energy method with the mathematical simulation of adjacent layers of the object under inspection [49].

In order to accelerate the detection of foreign materials in the object under inspection, it was proposed in [50] to scan objects simultaneously in two directions using two pairs of X-ray sources and detectors, while the sources could operate in a multi-energy mode (maximum radiation energy could change).

## 3. Technology Used for Results Analysis

Another important challenge in non-intrusive object inspection is the recognition of an object pattern and its classification aimed at differentiating legal, illicit, and dangerous objects. The most commonly used techniques in this field are related to machine learning (ML) ideas. According to its definition, machine learning is the study of computer algorithms that can improve automatically through experience and by the use of data [51,52]. In other words, ML could be described as the capability of a computer to learn how to solve a particular task without being explicitly programed with the use of a comprehensive solution algorithm. Traditionally, ML approaches are divided into three major categories, however, they could be extended:

(1) Supervised learning;
(2) Unsupervised learning;
(3) Reinforcement learning.

In supervised learning, a computer is fed with the set of labeled inputs in couples with their desired outputs (labels), given by a “teacher”, and the aim is to teach the computer the general rule for mapping inputs to outputs. After completing the training process, the derived mapping function or model will be later used to predict the output for new input data.

In unsupervised learning, the learning algorithms are fed with inputs that have no labels, so the algorithm is supposed to find the structure in the inputs on its own. An algorithm takes a set of input data trying to find correlations and structures in them such as grouping, clustering, or datapoints. So, these algorithms are not capable of responding to feedback, but identifying any commonalities in the input data instead, and detecting the presence or absence of these commonalities in any new piece of input data [53].

The intercategory approach, which uses both features of supervised and unsupervised learning, and is called a semi-supervised learning approach should also be mentioned. This approach uses a small amount of labeled data along with a number of unlabeled ones, which helps to significantly improve learning accuracy. Such a technique is typically used when it is not possible to obtain the necessary amount of training data for supervised learning, or the training labels are noisy, incomplete, or inaccurate.

Reinforcement learning algorithms that learn from experience, typically use the Markov decision process (MDP) to determine the future behavior of the system aimed at maximizing the performance of the specific task. This approach is mainly used in autonomous vehicles or for playing computer games with a human opponent where there is a necessity to dynamically predict system behavior based on experience.

To improve the learning experience, dimensionality reduction is widely used. This process aims to reduce the number of random variables under analysis by singling out only principal variables. Such an approach allows one to reduce the dimension of the features set, for example, by reducing high dimensional data to a smaller space (e.g., 3D to 2D), while keeping principal variables in the model [54,55,56,57]. With the help of machine learning tools, this approach could be implemented for developing data-driven models used for data classification based on processed X-ray images [58]. In particular, recent advances in autoencoder-based order reduction [59] are extremely promising in this context.

During the last decade, methods used to solve this task have significantly improved with the implementation of AI-based devices or techniques. The most common approach to items classification includes AI-training techniques with the subsequent implementation of pattern recognition and classification algorithms. All these techniques are successfully implemented in different parts of non-intrusive screening technology: for image recognition based on derived images from different types of X-ray machines; for other patterns recognition, such as air analysis to detect explosives and narcotic particles, and image analysis used in underwater object inspection.

### 3.1. Pattern Recognition

Pattern recognition is the process of automatic identification of individual patterns and regularities in data using machine learning (ML) algorithms [60]. Pattern recognition systems are capable of recognizing and classifying both familiar and unfamiliar objects, their shapes from different angles even if they are partially obscured or blurred. Using acquired data, this technique is also capable of providing the classification of identified patterns. In the case of supervised training, specially prepared or simulated labeled data are used for teaching the system to recognize the object of interest. In the case of unsupervised training, the system learns to detect similarities in unlabeled data structures [61]. The most popular tools used for pattern recognition include artificial neural networks [62,63,64], support vector machines [65,66], principal component analysis [67,68], multiple linear regression [69], generalized least square regression [70,71], linear discriminant, discriminant function, and stepwise discriminant analysis [71,72,73].

### 3.2. Support Vector Machines (SVM)

SVMs (also referred to as support-vector networks) represent themselves as supervised learning models, which could be implemented for classification as well as for regression analysis problems [74,75]. With this technique, a model is fed with a set of labeled input data, each one belonging to one or two specified categories. SVM training algorithm creates a model which maps training data samples to the point in a hyperspace aiming to maximize the gap between two different categories. Thus, to get the best fitting hyperspace for a certain category, the algorithm maximizes the distance between the closest points from different data classes, which are defined as support vectors. Such an approach allows one to categorize new input data as non-probabilistic input classifiers [75]. This technique is widely implemented in image classification and segmentation problems, demonstrating significantly higher accuracy compared to traditional query refinement schemes [76], in biological and other sciences to classify different objects, chemicals, and media. In particular, they are successfully used for odor recognition in EN [77,78,79,80].

### 3.3. Artificial Neural Networks (ANNs)

**Figure 7:**
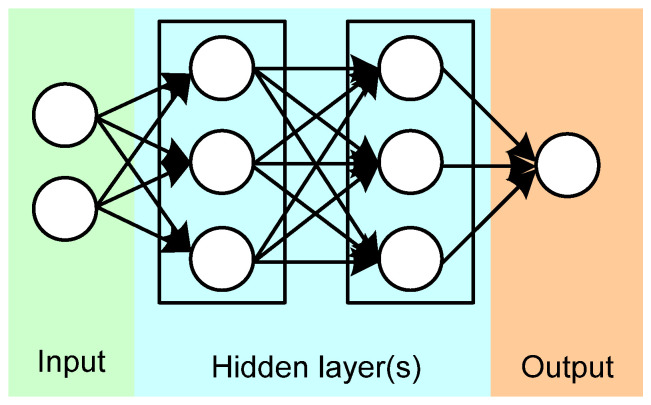
Typical structure of ANNs.

ANNs are computational frameworks based on the principles of biological neural networks operation inspired by animal brain functioning. ANN is a network of simple elements, called neurons, which takes information from their inputs, changes their inner state based on input (excitation), and produces output depending on input and excitation. The network is formed by interconnecting neuron output to other neuron inputs, resulting in the formation of an oriented weighted graph [81]. The typical structure of ANN is presented in Figure 7. Typically, ANNs have a layered structure, where different layers are responsible for different types of input conversion. Typically, ANN contains an input layer, which directly interacts with the external environment and is activated by consuming feeding information from its neuron inputs; then several hidden layers, which depend on the ANN architecture, and the outer layer, also directly interact with the external environment providing processed information from its neuron outputs [82]. Due to the capability of changing its internal structure depending on the changing external environment, along with provisioning output information dependent on the dynamically changing environment, ANN proved to be a good solution for the machine learning (ML) concept. Currently, they are widely implemented for classification problems, including image and sequence recognition, decision making, which is actively used in X-ray screening machines, as well as in EN applications [83]. However, the use of ANN assumes understanding peculiarities and differences in their architecture, including the ANN model itself, as well as teaching algorithm, and network robustness, which requires a large volume of datasets during the training process.

### 3.4. Deep Learning

Deep learning, also referred to as deep structured learning, is a branch of machine learning, which is based on a set of algorithms aiming to simulate higher level abstraction in datasets, implementing depth graphs with several processing layers built up with several linear or non-linear transformations. Deep learning architectures include recurrent neural networks [84], convolutional neural networks [85,86,87], deep belief networks, and others [88], including more sophisticated approaches such as generative adversarial networks (GANs) which can be effectively used to improve the resolution of the obtained images [89]. They could be applied to such fields, as drug design and recognition, bioinformatics, computer visioning, image recognition, and analysis [90,91,92,93], facial analysis [94,95,96,97,98], etc. Thus, it could be exploited for non-intrusive object inspection both in image recognition, classification, and analysis tasks [99,100,101,102], and in odor recognition techniques used for drug and explosives detection. The most typical technique used in deep learning for recognition and classification tasks usually employs convolutional neural networks as they provide automated feature extraction and selection, thus there is no need for preliminary preprocessing of input data [103]. As for recent applications, solutions capable of providing liquids and gas classification and identification should be mentioned [103,104,105], which could be also exploited for narcotics and explosives detection based on odor analysis.

Thus, it is concluded that nowadays techniques for automatic threat detection during non-intrusive object inspection are actively developing. Industrial security system manufacturers currently use several highly efficient techniques, but they are unavailable for mass use. On the other hand, there are attempts to develop inspection-aid systems, which implies cloud databases including a broad number of detected threats and illicit goods patterns, which could be used by different inspection organizations independently on the type of X-ray screening equipment.

However, such systems are trained only using visual and partially structural object recognition approaches, which do not always provide reliable results, especially during illegal object detection. Thus, it is necessary to employ additional techniques when there are doubts regarding the legitimacy of inspected objects to inspect as accurately, fast, reliable, and non-intrusively as possible. The pros and cons of the most used analysis methods are presented in Table 2.

## 4. Novel Approaches

While X-ray inspection currently is the most widely used non-intrusive technique, and it provides information about the shape and material of hidden objects, it still has limitations regarding detection and classification of a wide range of illicit and dangerous item types. Thus, a deeper inspection with the use of additional techniques might be needed, which could involve the use of specialized laboratory equipment or complex and expensive techniques which could not be easily accessible on sight at most of the control points, or even the need of manual inspection. This could entail non-desirable delays at the control points, which could be inconvenient for citizens and critical for businesses dependent on a huge trading flow. Thus, novel approaches for non-intrusive inspection, aimed at improving screening quality and speed, currently are under deep research and consideration.

### 4.1. Electronic Nose

One of the possible solutions is to employ knowledge gained from nature observation. Thus, it is commonly known, that for additional inspection specially trained bio-organisms, such as sniffer dogs, insects, or other sensitive animals, could be employed to detect certain materials, objects of phenomena (such as drugs, explosives, suspects, etc.) [106]. These methods have been proven to be highly effective; however, they are relatively slow, and bio-organisms are not able to perform their duties for a long period without rest. Typically, sniffing dogs could work for about one hour, and then they need to be rested [106]. Latest advances in microelectronics and informational technologies, along with deep cooperation between IT and biology specialists, have led to attempts to develop and deploy novel sensor types aimed at mimicking bio-organism sensing. This research could be divided into two big groups: the first group relies on the use of entirely technical sensors, and the second group uses so-called biosensors. Technical sensors are those which are made of only technical components, while biosensors include integrated biological elements. Technical sensors, in turn, could be divided into two groups: electronic noses, which contain various chemical sensors aimed at converting the detected chemicals into an analytic signal, and sensors based on instrumental analytics, such as mass gas chromatography, ion mobility spectrometry, etc. Biosensors contain bio-receptors (natural elements) that could use cells, proteins, cell tissue, or nanovesicles of real biological organisms [107]. The work is based on the detection of a biochemical reaction caused by contact with a measured substance and bioreceptor.

Refs. [107,108,109] analyze the possibility of employing electronic noses for solving real-life tasks, including a non-intrusive hidden object search aimed at detecting drugs and explosives based on air chemical analysis near the inspected object. Ref. [110] presents research results on the possibility of using electronic noses to detect explosive substances such as trinitrotoluene, cyclotrimethylenetrinitramine, and pentaerythritol tetranitrate by analysis of the relative concentration of these substances in the air.

**Figure 8:**
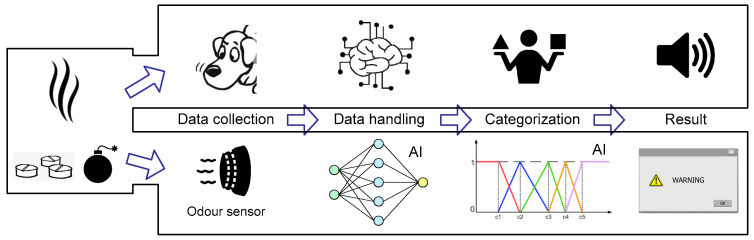
The typical odor recognition process.

A typical odor recognition process with the use of sensors could be represented as shown in Figure 8.

An odor sample is collected by a technical device or biosensor. Next, with the use of AI data analysis techniques (typically, CNN), the collected sample is analyzed detecting the most significant features for consequent analysis. Next, the prepared data are classified aiming to single out target patterns, such as drugs, explosives, etc. In case a suspected pattern is detected the systems produce an alarm signal.

As this technique is relatively novel, deep research is currently being carried out to improve the reliability and operability of these methods for practical implementation.

### 4.2. Mimicking Animal Sense Receptors

It is well known that the best ideas implemented in the most efficient technical products frequently were inspired by an observation of nature. It is well known that different animals have various ranges of specialized sensing organs dedicated to helping them in solving their everyday tasks crucial for surviving. Thus, bats employ echolocation to navigate and search for their prey. Mosquitos, bed bugs, and vampire bats can see a portion of ultrared vision and so on. Such abilities are observed by the bio-scientists and later adopted by engineering scientists in a piece of equipment for different possible purposes. Recently, several publications appeared related to adopting features of crocodile multisensory skin for different purposes [111,112], as their skin could sense mechanical, thermal, and chemical substances. These abilities were exploited by technical scientists to develop novel types of sensors that are able to more effectively and rapidly detect different features in objects under inspection [113]. One of the possible solutions is based on pressure and chemical analysis within the crocodile-inspired sensors, distinguishing hidden explosives.

### 4.3. Other Techniques

There is a number of other promising techniques for non-intrusive inspection based on spectroscopy, which are not widely used at the moment due to limitations in their construction, type of material which could be detected, high cost, or lack of experimental verification. Some of these methods are mentioned below.

The use of ultrasonic sensors to detect media inside tanks was proposed in [114]. This method was successfully tested to detect several types of explosive liquids and is currently under deeper research to widen the range of materials which could be detected.

A series of experiments were provided in [115] aiming to verify the effectiveness of the implementation of ultraviolet photovoltaic spectrography principle to detect certain type of explosives, such as TNT (2,4,6—trinitrotoulen), PETN (pentaerythritol), and AN (ammonium nitrate).

Another research proposed pharmaceutical material detection techniques through packaging with the use of spatially offset Raman spectroscopy [116]. This method could be implemented for drugs recognition, and the developed device could be portable.

Brief analysis showed that spectrography-based methods and techniques for non-intrusive inspection could be used as an auxiliary method to detect explosive substances or drugs.

## 5. Conclusions

In this review, we have presented and evaluated the most recent advances in non-intrusive inspection techniques, starting from human identity verification and ending with non-intrusive object inspection with the use of novel technologies, such as biosensors. The importance of improvement of the non-intrusive person and object inspection techniques is conditioned by increased human and trade flows, which could be used by intruders, smugglers, or even terrorists for moving illicit items or persons around the globe. Along with this, it was pointed out that it is highly desirable to improve the travelling experience for legitimate passengers, speeding up inspection processes at the control points.

To counter illicit person movements, different techniques for automated identity verification are provided in different states over the globe. Most of these techniques employ machine learning and other artificial intelligence techniques for facial recognition, and subsequent searches in worldwide interconnected databases using a person’s biological data. Such an approach assumes the deep interconnection of informational systems around the globe or at least around a certain area of the globe, which needs close cooperation among different state organizations from many countries. However, such complex systems could provide very fast identity verification (from several seconds up to several minutes), enhancing the travelling experience of legitimate passengers, while detecting illegal ones with a desirable tolerance level.

Another important issue is non-intrusive baggage or cargo inspection. In the paper, the most popular techniques that are currently being implemented and developed for this task, are reviewed. Nowadays, the most widely used techniques are those based on X-ray inspection. This group of techniques could provide detailed information on the shape and dimensions of the hidden object, and in some cases could be used for the determination of its material composition to detect organics, non-organics, and liquids. A detailed analysis of X-ray systems was provided, classifying their typical construction principle and the essence of their operation. It was pointed out, that even though X-ray systems with the ability to determine the material composition of an object, provide a wider range of object features, they are more expensive, and it is not economically reasonable to use them at every control point. Thus, the need to develop novel techniques and approaches was highlighted.

The most used techniques for X-ray machine results interpretation were analyzed. It was pointed out, that the most promising currently used approaches are those based on the use of the latest advances in computational technologies, mostly, the use of artificial intelligence techniques for recognition and classification tasks. The most widely used and promising AI techniques used in non-intrusive control were reviewed, focusing on novel promising techniques aimed at providing additional information or improving the operational and computational performance of existing ones.

Finally, novel approaches to non-intrusive inspection were analyzed. The most promising, from our point of view, are those which focused on the implementation of ideas observed from nature. Thus, a range of novel sensors based on animal sensing principles have appeared during the last few years. The most deeply investigated methods currently are those that use the principles of an animal smell sense to analyze odor composition. There are different approaches in such sensor construction: one group uses an entirely technical solution, while another group is focused on the use of natural elements (biosensors) to detect the media. The experimental results of the use of such types of sensors for odor recognition, proved their efficacy in detecting some types of explosive materials as well as drug types. Thus, the implementation of these solutions could be considered in the additional non-intrusive inspection process, for example, instead of sniffer dogs.

The review provided showed that even though existing techniques were successfully used to detect a wide range of threats during the inspection, there are still incidents of illicit trading, smuggling, and terrorism, as intruders also use the latest advances in techniques and digital technologies to bypass control points. Thus, there is strong demand for improving non-intrusive inspection techniques, which could be carried out with the use of novel sensor types, such as biosensors or animal-mimicking sensors. Furthermore, we argue that deploying interpretable deep learning methods [117] can significantly improve the performance of these applications.

## Figures and Tables

**Table 1:** Comparison of currently existent X-ray screening methods.

Technique	Shape	Density	Structure	Suspicious Substances
Planar radiography	possible, limitations while superpositioning	not possible	not possible	based on shape only [24,25]
X-ray computed tomography	clear, absence of superposition	possible, using multi-energy analysis	limited	based on shape and density analysis [26]
Dual- and multi-energy imaging	clear, absence of superposition	possible, using multi-energy analysis	limited	based on shape and density analysis [27,28,29]
Backscatter techniques	clear, absence of superposition	possible	possible	drugs, explosives, ceramic weapons [30,31]
X-ray diffraction imagining	clear, absence of superposition	possible	organics, non-organics, liquids	wide range of explosives, including crystalline, amorphous, liquid, home-made [32]

**Table 2:** Comparison of results analysis methods.

Method	Pros	Cons
Principle component analysis	Relatively fast due to data dimension reduction Could be applied for probability estimation for multi-dimensional data	Large computational time for huge datasets processing
SVM	Efficient for solving problems in multi-dimensional spaces Efficient in-memory consumption Rapid both for binary and multi-class classification Excellent result for nonlinear data processing Could be used for tasks with the number of data samples less than the number of dimensions	A relatively large computational time when a huge amount of data are processed Results significantly depend on noisy data which can lead to overlapping of classification classes
ANNs	Possible to apply for data with the incomplete initial knowledge High fault tolerance Could be implemented with parallel processing techniques Fast results after ANN being trained Efficient with the large datasets Could be applied both for regression and classification tasks	Hardware-dependent computational time Hard to find optimal network structure Overfitting may occur
Deep learning	Highly reliable recognition and classification results	Relatively more complex for training and implementation compared to other methods

## Data Availability

Not applicable.
